# Silicon Oxycarbide-Graphite Electrodes for High-Power Energy Storage Devices

**DOI:** 10.3390/ma13194302

**Published:** 2020-09-26

**Authors:** Dominik Knozowski, Magdalena Graczyk-Zajac, Grzegorz Trykowski, Monika Wilamowska-Zawłocka

**Affiliations:** 1 Department of Energy Conversion and Storage, Faculty of Chemistry, Gdańsk University of Technology, Narutowicza 11/12, 80-233 Gdańsk, Poland; dominik.knozowski@pg.edu.pl; 2 Fachbereich Material und Geowissenschaften, Technische Universität Darmstadt, Otto-Berndt-Straße 3, 64287 Darmstadt, Germany; graczyk@materials.tu-darmstadt.de; 3 EnBW Energie Baden-Württemberg AG, Fettweisstraße 44, 76189 Karlsruhe, Germany; 4 Faculty of Chemistry, Nicolaus Copernicus University in Torun, 87-100 Torun, Poland; tryki@umk.pl

**Keywords:** Silicon Oxycarbide (SiOC), graphite, composites, energy storage, lithium-ion capacitor (LIC)

## Abstract

Herein we present a study on polymer-derived silicon oxycarbide (SiOC)/graphite composites for a potential application as an electrode in high power energy storage devices, such as Lithium-Ion Capacitor (LIC). The composites were processed using high power ultrasound-assisted sol-gel synthesis followed by pyrolysis. The intensive sonication enhances gelation and drying process, improving the homogenous distribution of the graphitic flakes in the preceramic blends. The physicochemical investigation of SiOC/graphite composites using X-ray diffraction, ^29^Si solid state NMR and Raman spectroscopy indicated no reaction occurring between the components. The electrochemical measurements revealed enhanced capacity (by up to 63%) at high current rates (1.86 A g^−1^) recorded for SiOC/graphite composite compared to the pure components. Moreover, the addition of graphite to the SiOC matrix decreased the value of delithiation potential, which is a desirable feature for anodes in LIC.

## 1. Introduction

Over the past few years, there has been increasing interest in small high-power energy storage devices. For these applications electrochemical capacitors (ECs) are considered to be more suitable than conventional batteries. Unfortunately, ECs possess much lower energy density in comparison to batteries, namely 3–6 Wh kg^−1^ vs. 150–200 Wh kg^−1^, respectively [1,2], which severely limits their broader application. Many attempts have been made to enhance ECs energy density. Realizing an asymmetric system by employing a Faradaic, battery-like anode as a negative electrode is one of the most promising concepts. This idea was first presented by Amatucci in 2001 [3], and it is commonly known as an EC-battery hybrid or lithium-ion capacitor (LIC).

The main advantage of using a battery-like anode instead of the typical EC’s electrode is the extension of the overall potential difference. ECs operates from 0 to 2.7 V (in organic electrolytes) in charge/discharge processes, which results in diminishing potential difference between the electrodes during cycling and hence in limited energy density [4]. On the contrary, battery-like anodes hold constant potential during charge/discharge, which allows for keeping a higher potential difference over the entire cycling process. LIC preserves high power of EC thanks to processes separation—lithiation/delithiation occurs in anode and ${\mathrm{PF}}_{6}^{-}$ adsorption/desorption on cathode [5,6,7,8,9]. However, due to differences in electrochemical characteristics between electrodes, it is necessary to make proper electrodes matching in design.

On the anodic side, a few requirements must be met. The material should be characterized by a good conductivity, low lithiation/delithiation potential and provide sufficient capacity [3]. The capacity does not have to be exceptionally high, since it is often limited by the cathode [10,11], but at least 50 mAh g^−1^ should be available at potentials below 2 V vs. Li/Li^+^. The material should exhibit excellent rate capability to match the pace of cathodic reaction, and withstand polarization with 1–2 A g^−1^ for at least a few hundred cycles [11].

Having these requirements in mind, several solutions have been tested, specifically those originally applied in lithium-ion batteries. Various materials, based on Li_4_T_5_O_12_ (LTO) [3,12], transition metal oxides/graphene composites (Ni_2_O_5_, MnO_2_, CoO) [13,14,15], transition metal sulfides (MoS_2_, CoS) [16,17] or carbonaceous materials (hard carbon, soft carbon, graphite) [18,19,20] are reported as potential anodes for LIC. However, most of them, except for carbonaceous materials, exhibit a relatively high delithiation potential (approximately 1.5 V), which combined with low the capacity of a cathode does not provide high energy. Graphite exhibits very low lithiation potential: 0.005–0.25 V vs. Li/Li^+^. Moreover, graphite is abundant, non-toxic, has good cycle life and has a decent capacity for LIC applications. However, the performance of graphite in LIC is limited by its poor rate capability. Thus, many works have been focused on the improvement of the rate capability of graphite. To enhance the rate capability, various coatings [21], modifications of particle size, shape and porosity [22,23] or doping with various functional groups, nanoparticles or nanostructures have been reported [24,25]. Still, an unexplored solution is combining graphite with polymer-derived ceramics (PDCs). PDCs, including silicon oxycarbide (SiOC) and silicon carbon nitride (SiCN), are the materials consisting of SiC_x_O_y_ or SiC_x_N_y_ (x, y = 0–4) structural units, respectively, and a free carbon phase [26]. PDCs can be prepared by various routes, including liquid routes such as sol-gel, hydrosilylation or photocrosslinking, followed by a pyrolysis process. The liquid route allows facile mixing with other materials for making homogeneous composites. Pure PDCs have been tested as a material for lithium-ion batteries [27,28,29] and lithium-ion capacitors [30] as they exhibit high capacity up to 920 mAh g^−1^, good cyclability and good rate capability. PDCs have also been considered as an electrochemically active and mechanically resistant matrix for lithium-alloying compounds such silicon [31,32], tin [33,34] or antimony [35,36]. It has already been demonstrated by Kolb et al. [37] and Graczyk-Zajac et al. [38] that a combination with PDCs improves electrochemical properties of graphite. In this system, PDCs serve as a matrix, protecting graphite from deterioration. There are a number of works reporting the successful enhancement of materials’ energy storage properties via blending PDCs with carbonaceous materials, such as SiCN-graphite [37,38], SiCN-carbon nanotubes [39], SiCN-hard carbons [40,41], SiOC-carbon nanofibers [42], SiOC-CNTs [43] and SiOC-graphene [44]. However, to date there are still very few reports concerning SiOC-graphite composites and, to the best of our knowledge, no reports concerning the utilization of SiOC/graphite composite for LIC. The main advantage of SiOCs over SiCNs is the higher resistance of the preceramic polymer against oxidation, which facilitates material production [45,46].

In this work we investigate novel SiOC/graphite composites with phenyltriethoxysilane (PhTES) as the preceramic precursor. PhTES-based silicon oxycarbide exhibits high capacity and good rate capability [47]. We applied a new approach to blend the starting materials, namely, sol-gel synthesis enhanced by high-power ultrasounds. Sonication ensured good dispersion of graphite within the green body and accelerated gelation and drying processes. The new SiOC/graphite composite exhibited improved rate capability and high capacity in a lower potential range than pure SiOC, which makes it a promising candidate for negative electrodes in LIC.

## 2. Experimental Part

### 2.1. Synthesis of Composites

Silicon oxycarbide samples and the SiOC-based composites were synthesized by the sol-gel method, followed by pyrolysis. Phenyltriethoxysilane (PhTES) (>97%, Sigma Aldrich, Baden-Württemberg, Germany) was used as preceramic precursor. Different amounts of graphite powder (flakes, <20 µm, synthetic, Sigma Aldrich, Baden-Württemberg, Germany) were added to the synthesis solution to obtain SiOC/graphite composites of various carbon content. The synthesis procedure was as follows. First, graphite was poured into a three-neck flask. Graphite amount varied from 0 (pure ceramic sample—Denoted as SiOC_PhTES_), through 2, 4 and 10 g (composite samples denoted as SiOC_PhTES_/C2g, SiOC_PhTES_/C4g, and SiOC_PhTES_/C10g, respectively). Then, 17.3 g of PhTES and 6.6 g of absolute ethanol were added and carefully stirred. Next, 3.9 g of acidic water (pH = 4.5) were poured dropwise. Suspension was boiled for 1.5 h, then cooled down and transported into propylene test tubes. Afterwards, obtained sols were sonicated using high power homogenizer (UP200St, Hielscher Ultrasound Technology, Teltow, Germany) to obtain tar-like gel. Next, gels were dried for 5 days, with temperature increasing from 80 °C to 120 °C. The final step was pyrolysis at 1000 °C under argon atmosphere (heating ratio 100 °C/h, dwell time 1 h, constant Ar flow of 40 mL min^−1^).

### 2.2. Electrode Preparation and Electrochemical Measurements

Ceramic composites were ball milled (Mixer Mill, MM200, Retsch, Haan, Germany) to obtain fine powder. Then, powder was mixed with 10 wt.% solution of polyvinylidene fluoride (PVDF, Solef6020, Solvay, Rheinberg, Germany) dissolved in N-methyl 2-pyrrolidone (NMP) (BASF, Ludwigshafen, Germany) and Carbon Black Super P^®^ (Imerys Graphite & Carbon, Bodio, Swizerland). The ratio between powder, PVDF and carbon black was 85:10:5. In addition, 1–2 g of NMP were added for proper consistency. As received slurry was uniformly distributed onto a copper foil (10 μm, Copper SE-Cu58 Schlenk Metallfolien GmbH & Co. KG, Roth, Germany) using the doctor blade technique to obtain ~100 µm thick layers. Layers were then dried at 80 °C and punched into 10 mm discs. So, prepared disc electrodes were further dried at 80 °C under vacuum and transferred into a glove box (MBraun Glove Box Systems) where the cells were assembled.

For electrochemical testing Swagelok^®^ type two-electrode testing cells were assembled. In this system we used a disc electrode of the active material as a working electrode, a quartz filter paper MN GF-2 (45 µm, Macherey-Nagel GmbH & Co. KG, Roth, Germany) as a separator, a 1 M LiPF_6_ in 1:1 *v/v* ethylene carbonate:dimethyl carbonate (Sigma-Aldrich, Baden-Württemberg, Germany) as an electrolyte and a lithium foil (Sigma Aldrich, Baden-Württemberg, Germany) as a counter/reference electrode.

The materials were tested by cyclic voltammetry (CV) and galvanostatic charge–discharge (GCD) with potential limitation techniques. Electrochemical measurements were performed on the Biologic Potentiostat SP200 (BioLogic Science Instruments, Seyssinet-Pariset, France) while long-time cycle stability was conducted using a multichannel battery interface (Atlas 0961, Atlas Solllich, Rębiechowo, Poland). The potential window was set between 0.005 V and 3 V vs. Li/Li^+^ for both techniques. Materials were charged/discharged with the same current rate (*I*_charge_ = *I*_discharge_). Cyclic voltammetry was carried out with a scan rate of 0.1 mV s^−1^.

### 2.3. Characterization Techniques

Elemental analysis was conducted on a carbon analyzer (Leco C-200, Leco Corporation, St. Joseph, MI, USA) and a nitrogen/oxygen analyzer (Leco TC-436, Leco Corporation, St. Joseph, MI, USA). Carbon and oxygen content were measured directly while the silicon content was calculated as a complement to 100%. Thermal gravimetric analysis was performed on the SDT 2960 Simultaneous (TA Instruments, New Castle, DE, USA). X-ray diffraction spectra were obtained from the powder diffractometer Stoe STADI P equipped with a Mo Kα anode. Micro-Raman analysis within the wavenumber range of 100–3200 cm^−1^ was carried out using a confocal micro-Raman spectrometer (InVia, Renishaw, Wotton-under-Edge, UK) with an argon ion laser (514 nm). Raman spectra were deconvoluted using the OriginPro2016 software after the background subtraction. All the bands were fitted using Lorentzian peaks, except the D3 band, where a Gaussian curve was used. XPS analysis was carried out on the Escalab 250Xi spectroscope (Thermo-Fischer Scientific, Waltham, MA, USA) with a monochromatic Al Kα source. The morphology was characterized by a transmission electron microscope TEM (FEI, G2 F20X-Twin 200 kV, FEG, Hillsboro, OR, USA). Energy-dispersive X-ray spectroscopy EDS (EDAX, RTEM model SN9577, 134 eV) was used to identify the chemical elements in designated areas. Measurements were made in the TEM mode (bright-field) and the STEM mode (HAADF and EDX detectors). Preparation of the samples was as follows: a few milligrams of the powder were dispersed in ethanol (99.8% anhydrous) with the aid of ultrasounds for 5 s, and a drop of the dispersion (5 mL) was applied on a carbon-coated copper mesh with holes (Lacey type Cu 400 mesh, Plano, TX, USA), and stored in the room temperature until the complete evaporation of solvent. MAS-NMR measurements were performed on the Bruker Avance Ultrashield 500 MHz spectrometer. ^29^Si NMR spectra were recorded with the following parameters: single pulse sequence, ^29^Si frequency: 139.11 MHz, π/8 pulse length: 2.5 ms, recycle delay: 100 s, 1k scans, external secondary reference: DSS. 3.2 mm zirconia rotors filled with samples were spun at 8 kHz under air flow.

## 3. Results and Discussion

**Figure 1:**
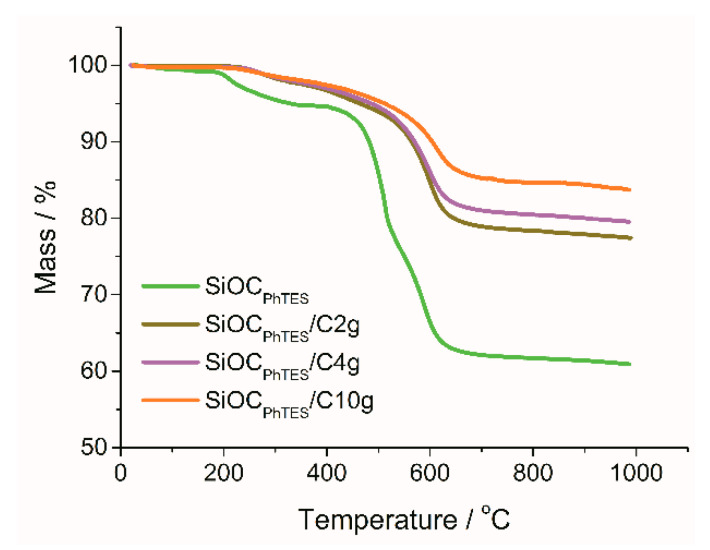
Dependence of the mass yield on the temperature of the SiOC/graphite composites.

Figure 1 presents the thermographs of the investigated materials, i.e., SiOC_PhTES_, SiOC_PhTES_/C2g, SiOC_PhTES_/C4g, and SiOC_PhTES_/C10g. The analysis provides information about mass loss during the pyrolysis process. It shows the influence of graphite addition on a thermal conversion yield of polymer/graphite blends. Pure SiOC_PhTES_ exhibited two significant mass losses: the first one between 50 °C and 350 °C, and the second one in the temperature range of 400–600 °C. The first one is attributed to the release of by-products of the polycondensation reaction and the residual EtOH/H_2_O. The second one is related to the mass loss occurring during the redistribution reaction, i.e., the exchange of Si-O bonds with Si-H and/or Si-C bonds, with the simultaneous release of volatile compounds, mainly H_2_, CH_4_ or C_2_H_2_ [48,49]. The overall ceramic yield of SiOC_PhTES_ was around 60.9%, which stays in accordance with the literature [47,50]. When graphite was added to the material, we observed a proportional increase of the mass yield during pyrolysis. The mass yield of SiOC_PhTES_/C2g, SiOC_PhTES_/C4g and SiOC_PhTES_/C10g was equal to 77.4%, 79.5% and 83.7%, respectively. This is mostly related to a smaller mass loss in the temperature range of 400–500 °C, which is a consequence of a lower PhTES content in the preceramic polymer/graphite blends. These results indicate that the content of graphite in the preceramic matrix did not change during pyrolysis in an argon atmosphere, and the final mass yield was directly related to the starting material composition.

The TGA results stay in agreement with the results of the elemental analysis shown in Table 1. As expected, the increase in the amount of graphite in the preceramic blends led to a higher amount of carbon in a final ceramic composite. Free carbon content in the pure SiOC_PhTES_ ceramic, calculated using the approach of Soraru et al. [51], was 34.2 wt.%, which represents 91% of the total carbon in the sample. For the SiOC/graphite composites, the free carbon phase constituted from 95% (for SiOC_PhTES_/C2g) to almost 100% (for SiOC_PhTES_/C10g) of the total carbon.

**Figure 2:**
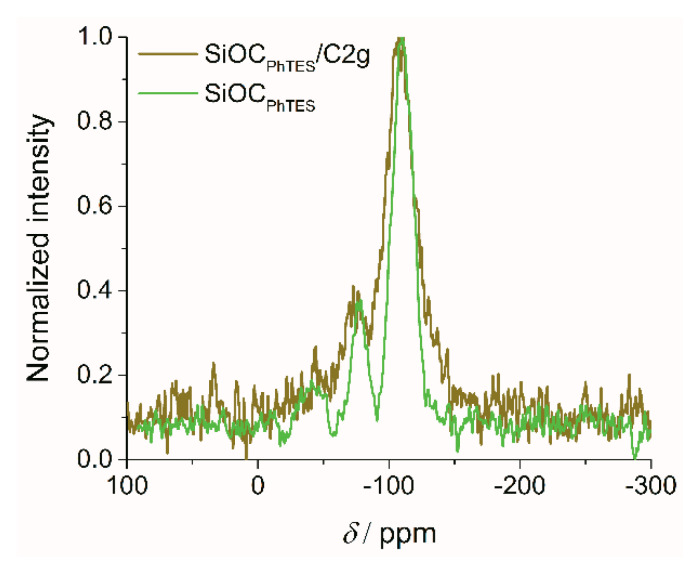
^29^Si MAS-NMR spectra of the SiOC_PhTES_ and SiOC_PhTES_/C2g samples.

^29^Silicon Solid-state NMR measurements (^29^Si MAS-NMR) were conducted in order to determine the change in the redistribution of various SiO_x_C_y_ tetrahedra units resulting from the addition of graphite. Figure 2 shows the corresponding ^29^Si MAS-NMR spectra of SiOC_PhTES_ and SiOC_PhTES_/C2g. The ^29^Si MAS-NMR spectrum of the SiOC_PhTES_/C2g composite exhibits broader peaks at approximately −109, −73 and −48 ppm, and higher noise than the spectrum of the pure SiOC_PhTES_ ceramic sample. However, the share of SiO_4_ and mixed bonds SiO_3_C, SiO_2_C_2_ tetrahedra is comparable for both samples (Table 2 and ref. [47]). SiO_4_ tetrahedra dominate in both samples (76%–78%), and the mixed bonds constitute from several up to over a dozen percent. This suggests that there was no reaction between the preceramic polymer and graphite at any of the stages of preparation of the composites, namely during hydrolysis and condensation reactions, high-power ultrasound-assisted gelation process, nor pyrolysis at 1000 °C.

XPS results (presented in Supplementary Materials—SM, Figure S1) confirm the collected NMR data. XPS Si2p spectra of pure ceramic and the composite samples look almost the same. A broad peak fitted with the Si2p_3/2_ and Si2p_1/2_ doublet at binding energies of 103.2–103.4 and 103.7–103.8 eV, respectively, corresponding to SiO_4_ tetrahedra [52,53] is observed. On the other hand, the C1s spectra of the pure ceramic sample and SiOC/graphite composite show some differences in the share of particular bonds. Both C1s spectra (Figure S2) were deconvoluted with four peaks at BE: 284.0–284.4 eV, 285.2–285.4 eV, 286.5–286.7 eV and 288.8–289.2 eV, which may be attributed to C-Si/C=C, C-C/C-H, C-O and C=O bonds, respectively [54,55,56,57]. However, the C1s spectrum of the SiOC/graphite composite shows a significantly larger peak corresponding to the C-C bond, and smaller peaks assigned to the C-Si, C-O and C=O bonds, which is related to a high graphite content in the composite.

**Figure 3:**
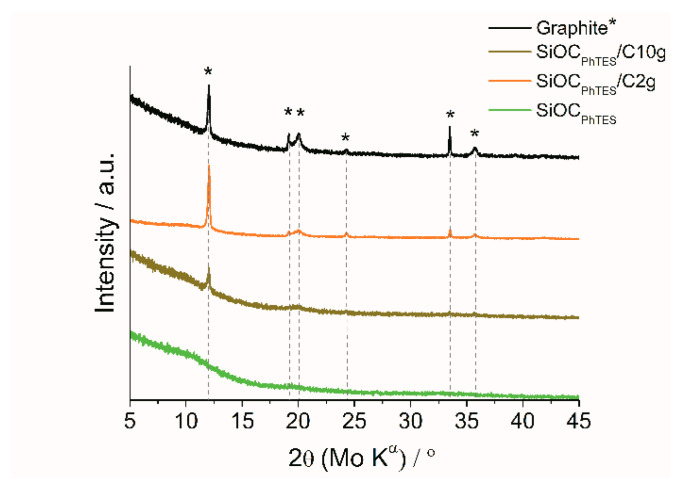
Diffractograms of pure graphite, ceramic and SiOC/graphite composites. * is in the legend of the graph, meaning the diffraction peaks for graphite.

The homogenization method applied for blending of the materials requires high power ultrasound, which can have a destructive effect on the graphite structure. To evaluate possible changes in the graphite lattice XRD measurements were performed. Figure 3 shows the diffractograms of pure graphite, ceramic, and SiOC_PhTES_/graphite composites. For the graphite sample sharp peaks at 12°, 19.1°, 20.1° and 33.5° (2θ Mo Kα, which correspond to 26.2, 42.2, 44.5 and 77.4 for Cu Kα) can be distinguished, described in literature as [2], [100], [101] and [110] Bragg peaks of typical hexagonal graphite [58,59]. In contrast, pure SiOC_PhTES_ diffractograms show only a broad halo typical for amorphous materials [51,60], while for the SiOC/graphite composites the peaks typical for graphite appear. The higher amount of graphite in the composite, the more pronounced reflexes in the diffractograms are detected. The diffractogram of SiOC_PhTES_/C10g exhibits sharp peaks, which indicate that the skeleton of the graphite structure was preserved despite the usage of high-power ultrasounds during the synthesis.

**Figure 4:**
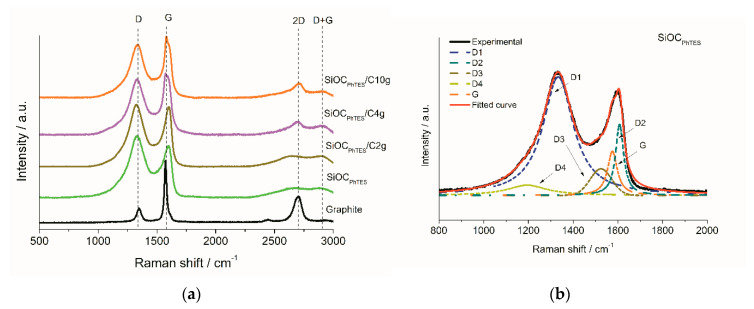

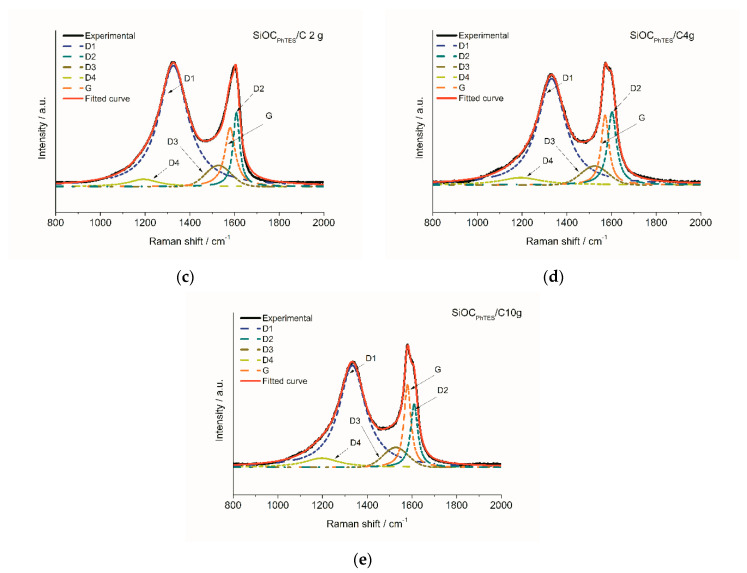
(**a**) Comparison of Raman spectra recorded for investigated samples. Fitting of the Raman spectra of: (**b**) SiOC_PhTES_, (**c**) SiOC_PhTES_/C2g, (**d**) SiOC_PhTES_/C4g and (**e**) SiOC_PhTES_/C10g samples.

More detailed analysis of the carbon microstructure was performed by means of Raman spectroscopy. Figure 4a depicts Raman spectra of the investigated samples recorded in the range of 500–3000 cm^−1^ after the background subtraction. Two characteristic bands in the first-order spectra, namely the D-band at approximately 1333 cm^−1^, and the G-band at 1575 cm^−1^, representing disordered and graphitic carbon, respectively, appear for all of the investigated composites. In order to perform a quantitative analysis of the measured spectra, we deconvoluted the first-order Raman signals into five peaks (Figure 4b–e), namely D1, D2, D3, D4 and G according to [61,62].

In the spectra of the pure ceramic and the composites samples, the D band is separated into a main D1 peak and a small shoulder marked as D4. Both peaks, i.e., D1 and D4, result from a graphitic lattice vibration mode with A_1g_-symmetry, which is typical for disordered carbons. D1 is assigned to graphene edges [63,64,65] while the D4 is related to Csp^2^-Csp^3^ bonds [66] or ionic impurities [67]. The G band was deconvoluted into a G peak, arising from stretching vibrations of the sp^2^-carbon bond in the ideal graphitic lattice (E_2g_ symmetry), and a D2 peak, related to the defected graphitic lattice (also E_2g_ symmetry) [61,64,65] and Stone–Wales defects [68], which is also present in the deconvoluted spectrum of the pure graphite sample (Figure S3a in Supplementary Materials). Moreover, between the D1 and G bands another band, namely a D3 band at ~1525 cm^−1^, sp^2^ amorphous forms of carbon [61,62], is detected. Detailed information on band parameters is given in Table 3 and Table S1 in the Supplementary Materials.

With the increasing amount of graphite in the composites one can notice a significant rise in the G band intensity, from 0.36 for the pure ceramic SiOC_PhTES_ to 0.68 for SiOC_PhTES_/C10g, along with a decrease in the intensity of the D1 and D4 bands, from 0.98 to 0.84 and from 0.084 to 0.075 for these materials, respectively. These differences are even more pronounced when we compare the *I*_D1_/*I*_G_ and *I*_D2_/*I*_G_ intensity ratios that decreased with the increasing graphite content in the ceramic matrix. The *I*_D1_/*I*_G_ ratio dropped from 2.69 for the pure ceramic to 1.24 for the SiOC_PhTES_/C10g sample, whereas *I*_D2_/*I*_G_ decreased from 1.61 to 0.76. Moreover, composites with graphite exhibit lower intensity of the D3 band compared to the pure ceramics. These results prove that the addition of graphite to the composites increased the content of the ordered carbon phase in the material, suggesting that the graphitic structure was hardly affected by the ultrasound treatment.

Furthermore, we tested the influence of ultrasounds on the size of graphite flakes used for the synthesis of the composites. In that case, we immersed graphitic powder into test tubes with isopropanol and subjected it to ultrasounds for a time of up to 2 h (analogous to the time used for composite blending). After drying, the morphology of the graphite powder was examined by SEM and no changes with respect to the pristine graphitic morphology were identified (see SEM Figure S4a,b, Supplementary Materials). This suggests that the structure of graphite was preserved in the composites.

In the second-order Raman spectra, the bands at 2700 cm^−1^ (2D) and 2900 cm^−1^ (D + G) were detected, i.e., the overtones of D-band along with combined D and G bands, respectively. The 2D band correlates with the number of stacked carbon layers that make up graphite clusters. The second-order Raman spectra are presented in more detail in Figure S3b (Supplementary Materials). Graphite exhibits a very pronounced 2D peak at 2702 cm^−1^, indicating stacked undamaged graphene layers. In contrast, the SiOC_PhTES_ shows an asymmetric and blurred 2D peak, indicating high disorder in the carbon structure. With increasing graphite content in the composites, the 2D band becomes narrower and more intense, signifying increased content of a more ordered carbon phase. The overall Raman results do not indicate any damages in the graphitic structure provoked by the ultrasound-assisted synthesis method, confirming the results obtained by NMR and XRD analysis.

**Figure 5:**
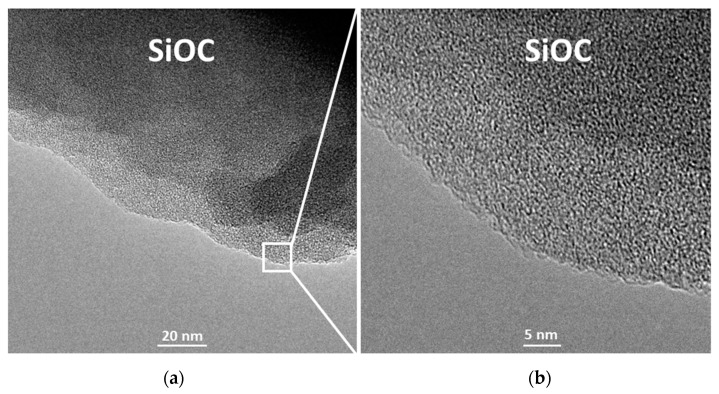

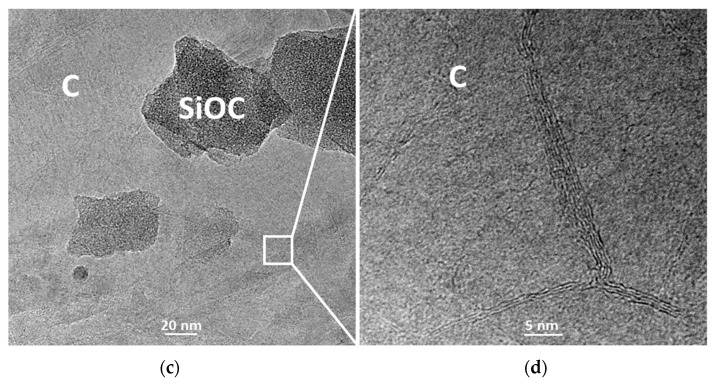
TEM images of: (**a**), (**b**) SiOC_PhTES_ and (**c**), (**d**) SiOC_PhTES_/C10g samples.

TEM imaging was performed in order to investigate more deeply the microstructure of the composites. Figure 5a,b show TEM images of the amorphous organization of SiOC_PhTES_ sample, (typical for pure silicon oxycarbide pyrolyzed at 1000 °C). On the other hand, two separate phases, representing the amorphous SiOC and the more ordered carbon material, are observed for the SiOC_PhTES_/C10g sample (Figure 5c,d).

**Figure 6:**
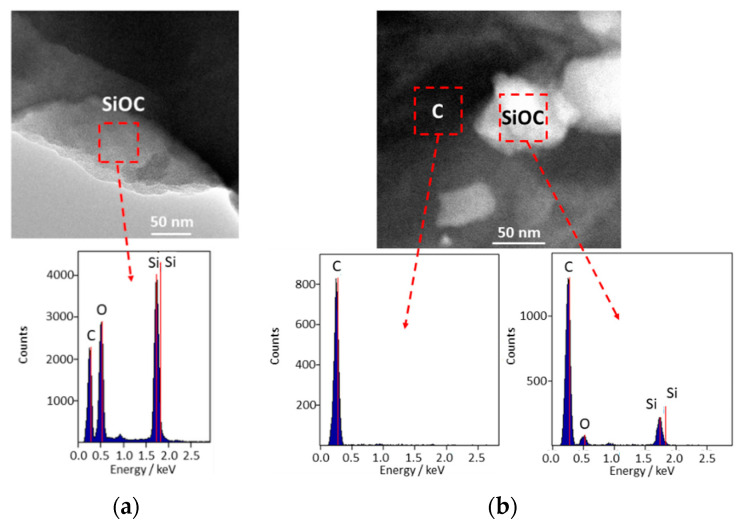
TEM images of: (**a**) SiOC_PhTES_ and (**b**) SiOC_PhTES_/C10g samples.

STEM-EDX measurements confirm the presence of a uniform silicon oxycarbide phase for the SiOC_PhTES_ sample, and two separate phases for the SiOC_PhTES_/C10g composite one, where only carbon is identified, and the second one, where carbon, silicon and oxygen dominate (Figure 6a,b).

## 4. Electrochemical Testing

**Figure 7:**
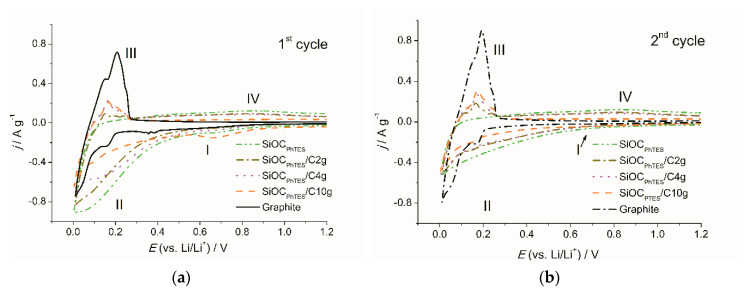
Cyclic voltammetry curves of the investigated materials: (**a**) first cycle, (**b**) second cycle; scan rate 0.1 mV s^−1^.

Figure 7a,b show CV plots obtained for the SiOC_PhTES_/graphite composites and pure components for comparison. During the first cycle, all the composites and pristine ceramic materials exhibited a small cathodic peak at 0.7 V (marked as (I) on the plot), which disappear in the following cycles. This corresponds to the formation of the solid-electrolyte interface (SEI) on the boundaries of the ceramic phase [69,70]. Further on the CV curves of the SiOC-based materials, a broad peak between 0 and 0.3 V (II), which corresponds to lithium insertion into the ceramic [29,69,71], is present. The cathodic current in this range decreases with the increasing graphite content in the composites, but there are no pronounced peaks at 0.16 and 0.05 V as registered for pure graphite. The intensity of the peak (II) is dropping in the following cycles (Figure 7b), which corresponds to the stabilization of the lithium insertion [72]. On the anodic site of the CV of the composites, we observe peaks between 0 and 0.25 V (III) corresponding to graphite delithiation [73] and a broad plateau-like peak (IV) characteristic for a typical lithium extraction from the ceramic [71]. The intensity of the peaks observed on CV curves depends on the composition of the material and follows the trend of increasing graphite content. For graphite-rich composites, one may identify more pronounced peaks (III), while for graphite-poor composites the peak (IV) is more intense.

**Figure 8:**
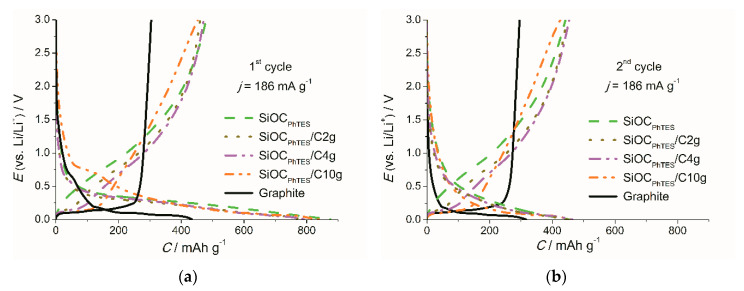
Galvanostatic charge–discharge curves of the investigated materials recorded at the current density (j) of 186 mA g^−1^: (**a**) first, (**b**) second cycle.

The capacity distribution over the potential range of 0–3 V and the Coulombic efficiencies were assessed from charge/discharge profiles. Figure 8a,b show charge/discharge curves of the first and second cycles, respectively, while more detailed information is presented in Table 4. The first cycle lithiation profiles of the SiOC_PhTES_, SiOC_PhTES_/C2g and SiOC_PhTES_/C4g samples are very similar. A different curve shape with a quasi-plateau at around 0.7 V, related to SEI formation, is noticed for the SiOC_PhTES_/C10g sample. Galvanostatic charge–discharge curves correspond well to the cyclic voltammetry curves. The trends of increasing length of the plateau, corresponding to delithiation of the graphitic phase, and a decrease in the delithiation potential with increasing graphite content in the ceramic matrix can be identified. A low delithiation potential is one of the features expected for LIC anodes. The curves of the SiOC_PhTES_/C2g and SiOC_PhTES_/C4g samples exhibit small plateaus in the 0.11–0.15 V voltage range, followed by a more rapid voltage increase, while the plateau recorded for the SiOC_PhTES_/C10g composite is significantly longer, and the onset of the faster voltage rise is observed at 0.2 V. This results in the higher capacity recovered below 0.5 V for the SiOC_PhTES_/C10g sample compared to the composites and the pure ceramics.

The electrochemical contribution of both components, namely SiOC and graphite, is unambiguously exposed on charge–discharge curves of the investigated composites, and the contribution of each part is proportional to its content. For graphite-poor samples, a small plateau originating from graphite, and a long ascending curve typical for ceramics can be noticed, while in the case of graphite-rich sample a much larger plateau is observed.

**Figure 9:**
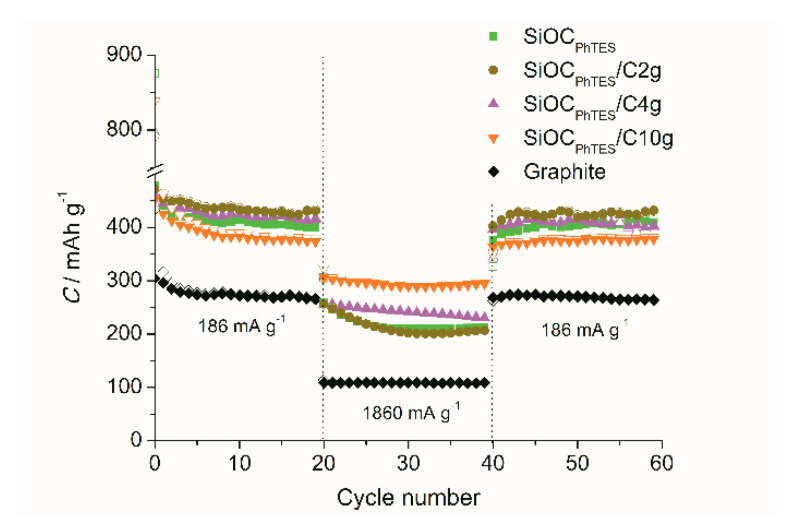
Lithiation (empty symbol) and delithiation (solid symbol) capacity upon extended cycling at C/2 and 5C rate for the investigated materials.

The charge/discharge profile allows one to evaluate the Coulombic efficiency of the material. One could expect that the first cycle efficiency (FCE) should increase with the addition of graphite to the composites. However, the graphite flakes used for composite preparation exhibit relatively low Coulombic efficiency of the first cycle (lower than reported by electrode suppliers [74,75] or in literature [76,77]). This may be caused by lower crystallinity and higher surface area of the flakes than of graphite used in commercial batteries. Addition of small quantities of graphite seems to slightly increase the FCE (59.6% and 57.8% for the SiOC_PhTES_/C2g and SiOC_PhTES_/C4g, respectively, compared to 54.7% for the pure ceramic). However, the sample with the highest graphite content, i.e., the SiOC_PhTES_/C10g, showed the FCE of only 53.9%. This may be caused by higher activity at 0.7 V observed on the CV and GCD curves during the first cycle. In the following cycles, all studied materials show efficiency of over 99%. Extended cycling of the electrodes at the C/2 and 5C current rates (C = 372 mA g^−1^) is presented in Figure 9. All the SiOC-based materials show better electrochemical performance than graphite. The difference is even more pronounced at high current rates. Note that for the C/2 rate the highest capacity is recorded for the composite with the lowest graphite content (423 mAh g^−1^ for SiOC_PhTES_/C2g), while for the 5C rate, the graphite-rich composite has the highest capacity (293 mAh g^−1^ for SiOC_PhTES_/C10g compared to 218 mAh g^−1^ for the pure ceramic SiOC_PhTES_ and only 108 mAh g^−1^ for the graphite electrode). High capacity is another feature of anodes that are suitable for high power energy devices. Better electrochemical performance of the SiOC_PhTES_/C10g sample compared to the SiOC_PhTES_ one may follow from the carbon content in these materials. According to elemental analysis (Table 1), the amount of carbon increases from 37.4 wt.% for pure ceramic to 68.7 wt.% for the composites with the highest graphite content. A higher amount of carbon in the ceramic matrix leads to a higher electronic conductivity and a larger number of diffusion paths for lithium ions, crucial upon polarization with high currents. Slightly lower capacity showed by the SiOC_PhTES_/C10g composites compared to the pure ceramic at the C/2 rate is probably due to a lower share of the active sites for lithium storage present in the ceramics [78]. Low capacity of pure graphite at a high current rate is often explained by material exfoliation caused by electrolyte penetration along with lithium ions [79].

**Figure 10:**
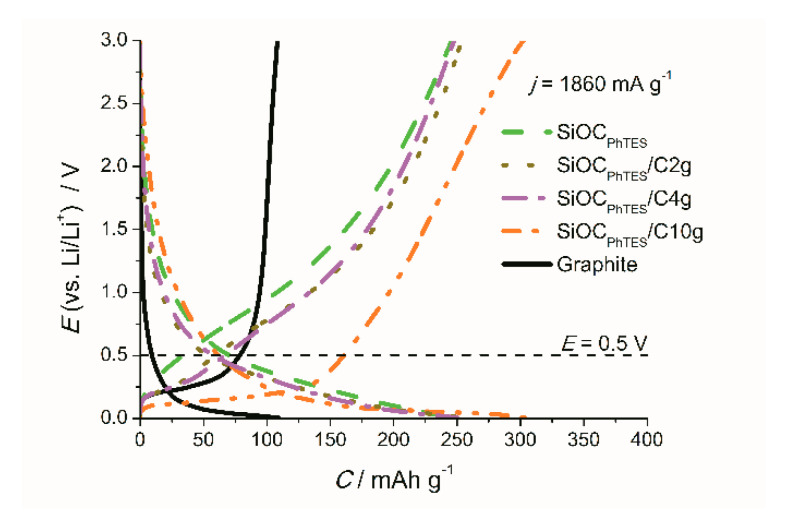
Galvanostatic charge–discharge curves of the investigated materials recorded at 1860 mA g^−1^.

To examine the phenomenon of high capacity at high currents for graphite rich composite, we took a closer look at the charge/discharge profiles collected upon polarization at a 5C rate (Figure 10). GCD curves presented in Figure 10 were recorded after 20 cycles at a C/2 rate in order to show a stable response of the electrodes with Coulombic efficiency of over 99%. The shape of the composite curves at 5C exhibits the same tendency as the curves recorded at a C/2 rate (more extended plateau and a lower delithiation voltage for graphite-rich samples), except for the capacity value, which is the highest for the SiOC_PhTES_/C10g electrode. These features make the material promising for the application in LIC. What is also essential for the potential application in LIC, is that this composite delivers the highest capacity below 0.5 V.

In Table 5, the capacity values of the most popular potential anodes for LIC reported in the literature are presented. The focus was on the capacity measured at a 2 A g^−1^ current rate typical for testing of LIC [4], and voltage range below 0.5 V. To the best of our knowledge, the SiOC_PhTES_/C10g composite exhibits the highest capacity upon these conditions among all popular LIC anode materials. Our composite exhibits twice as high capacity as the competitive materials. Considering the potential range (0–3 V), our composite also shows a satisfactory capacity of almost 300 mAh g^−1^.

## 5. Conclusions

In this work, we evaluated various graphite-ceramic based composites with different graphite content for potential application as anodes in LIC. The materials were prepared by a novel method utilizing high power ultrasounds. Sonication facilitates the gelation process and uniform distribution of graphite flakes within the preceramic polymer. Silicon oxycarbide is an electrochemically active component contributing to the capacity of composites and plays the additional role of a matrix for graphitic flakes, causing the stabilization of the electrochemical response at high current rates. Moreover, the addition of graphite to SiOC shifts lithiation and delithiation processes towards lower potentials in comparison to the pure SiOC. The best performing material seems to be the SiOC_PhTES_/C10g one, i.e., the composite with the highest investigated graphite content. This material is characterized by a high capacity of 294 mAh g^−1^ at a 5C current rate, among which over 160 mAh g^−1^ is recovered below 0.5 V vs. Li/Li^+^. This makes the SiOC_PhTES_/C10g material a potential candidate for anodes used in high power energy storage devices, e.g., lithium-ion capacitors.

## Figures and Tables

**Table 1:** Results of elemental analysis of SiOC_PhTES_ and SiOC_PhTES_/graphite composites.

Material	C	O	Si	C_free_	Empirical Formula
	wt.%				
SiOC_PhTES_	37.4	29.3	33.3	34.2	SiO_1.546_C_2.632_
SiOC_PhTES_/C2g	51.3	22.9	25.8	48.7	SiO_1.537_C_4.604_
SiOC_PhTES_/C4g	55.9	22.5	21.6	55.1	SiO_1.825_C_6.055_
SiOC_PhTES_/C10g	68.7	16.6	14.7	68.5	SiO_1.968_C_10.877_

**Table 2:** Data obtained from fitting of ^29^Si MAS-NMR spectra of the pure ceramic and the composite samples.

Sample	SiO_4_		SiO_3_C		SiO_2_C_2_	
	δ/ppm	%	δ/ppm	%	δ/ppm	%
SiOC_PhTES_^a^	−104.6	76.0	−69.9	16.4	−22.1	0.6
					−38.2	7.1
SiOC_PhTES_/C2g	−109.0	78.2	−73.0	11.0	−48.8	10.8

^a^ results from our previous work [47].

**Table 3:** Data obtained from the Raman spectra deconvolution of the investigated samples, band positions, band intensities (*I*) (height of the fitted peak) and intensity ratio (*I*_D1_/*I*_G_ and *I*_D2_/*I*_G_).

Material	D4		D1		D3		G		D2		*I*_D1/_ *I*_G_	*I*_D2/_ *I*_G_
	cm^−1^	*I*_D4_	cm^−1^	*I*_D1_	cm^−1^	*I*_D3_	cm^−1^	*I*_G_	cm^−1^	*I*_D2_		
SiOC_PhTES_	1194	0.084	1333	0.98	1525	0.22	1575	0.36	1608	0.58	2.69	1.61
SiOC_PhTES_/C2g	1193	0.06	1327	1	1528	0.17	1580	0.48	1609	0.61	2.06	1.26
SiOC_PhTES_/C4g	1195	0.059	1332	0.88	1525	0.16	1571	0.57	1603	0.6	1.53	1.05
SiOC_PhTES_/C10g	1197	0.075	1333	0.84	1528	0.16	1580	0.68	1610	0.52	1.24	0.76
Graphite	-	-	1348	0.22	-	-	1573	0.97	1611	0.04	0.22	0.04

**Table 4:** Reversible C_rev_ and irreversible C_irrev_ capacity values of the first cycle upon polarization with 0.186 A g^−1^, Coulombic efficiency of the first cycle *η* and the average delithiation capacities CD of graphite, ceramic and composite samples measured at different current rates (average capacity values calculated from data presented in Figure 9).

Sample	1st Cycle C_rev_/mAh g^−1^	1st Cycle C_irrev_/mAh g^−1^	*η*	Average CD	
				1.86 A g^−1^	0.186 A g^−1^
Graphite	304	129	70.2	108	268
SiOC_PhTES_	479	396	54.7	218	402
SiOC_PhTES_/C2g	472	320	59.6	214	423
SiOC_PhTES_/C4g	460	336	57.8	242	408
SiOC_PhTES_/C10g	452	386	53.9	293	372

**Table 5:** Capacity recovered below 0.5 V at high current rates for LIC anodic materials.

Material	Counter Electrode	Current Rate/A g^−1^	Capacity in the Voltage Range		Ref.
			0–0.5 V/mAh g^−1^	0–3 V/mAh g^−1^	
MnO_2_/graphene aerogel	Li metal	2	~60	257	[14]
TiC accordion	AC^a^	1.3	~50	65 (0–2.5 V)	[80]
TiNb_2_O_7_ nanorods	Li metal	1.9	0^b^	225	[81]
CTAB-Sn(IV)@Ti_3_C_2_	Li metal	1	~40	~480	[82]
Nb_2_O_5_ nanorods film	Li metal	2	0^b^	154	[83]
LTO-graphene	Li metal	1.75	0^b^	180	[84]
N-doped graphene sheet	Li metal	2	~30	205	[85]
MnFe_2_O_4_/carbon	Li metal	2	~65	~600	[86]
BiVO_4_ nanorods	Li metal	2.3	~70	~700	[87]
Fe_3_O_4_@carbon	Li metal	2	~10	~587	[88]
SiOC	Li metal	0.2	~90	238 (0–1 V)	[30]
Graphite	Li metal	1.9	79	108	Our work
SiOC_PhTES_	Li metal	1.9	34	246	Our work
SiOC_PhTES_/C10g	Li metal	1.9	161	294	Our work

AC^a^—Activated carbon; ^b^ measurements started above 0.5 V, so we assume negligible capacity below 0.5 V. “~” sign appears for the values assessed from the graph presented in the cited paper.

## Supplementary Materials

The following are available online at https://www.mdpi.com/1996-1944/13/19/4302/s1, Figure S1: XPS Si2p spectra of (a) SiOC_PhTES_ and (b) SiOC_PhTES_/C10g samples., Figure S2: XPS C1s spectra of (a) SiOC_PhTES_ and (b) SiOC_PhTES_/C10g samples., Figure S3: (a) Deconvolution of Raman spectra of the pure graphite, (b) second-order Raman spectra of graphite, ceramic and SiOC/graphite composites, Figure S4: pictures of (a) graphite flakes, (b) graphite flakes after 2 h sonication in isopropanol, Table S1: Data obtained from the deconvolution of Raman spectra.
