# Supplementary material for: Silicon Oxycarbide-Graphite Electrodes for High-Power Energy Storage Devices
Source: Materials (Basel). 2020 Sep 26;13(19):4302. doi: 10.3390/ma13194302 (PMC7578959; doi:10.3390/ma13194302)
Supplement: Supplementary file 1 [file materials-13-04302-s001.docx]

Supplementary Materials

Silicon Oxycarbide-Graphite Electrodes for High-Power Energy Storage Devices

Dominik Knozowski ^1^, Magdalena Graczyk-Zajac ^2^, Grzegorz Trykowski ^3^ and
Monika Wilamowska-Zawłocka ^1,^*

^1^ Department of Energy Conversion and Storage, Faculty of Chemistry, Gdańsk University of Technology, Narutowicza 11/12, 80-233 Gdańsk, Poland; dominik.knozowski@pg.edu.pl

^2^ Fachbereich Material und Geowissenschaften, Technische Universität Darmstadt, Otto-Berndt-Straße 3, 64287 Darmstadt, Germany; graczyk@materials.tu-darmstadt.de

^3^ Faculty of Chemistry, Nicolaus Copernicus University in Torun, 87-100 Torun, Poland; tryki@umk.pl

***** Correspondence: monika.wilamowska@pg.edu.pl; Tel.: +48-58-347-24-74

Received: 01 September 2020; Accepted: 23 September 2020; Published: date

| 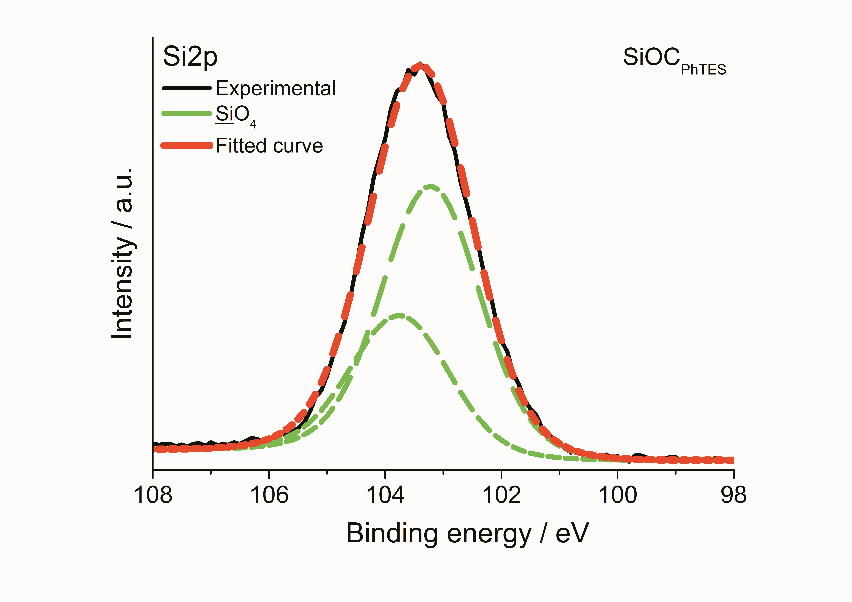  (**a**) | 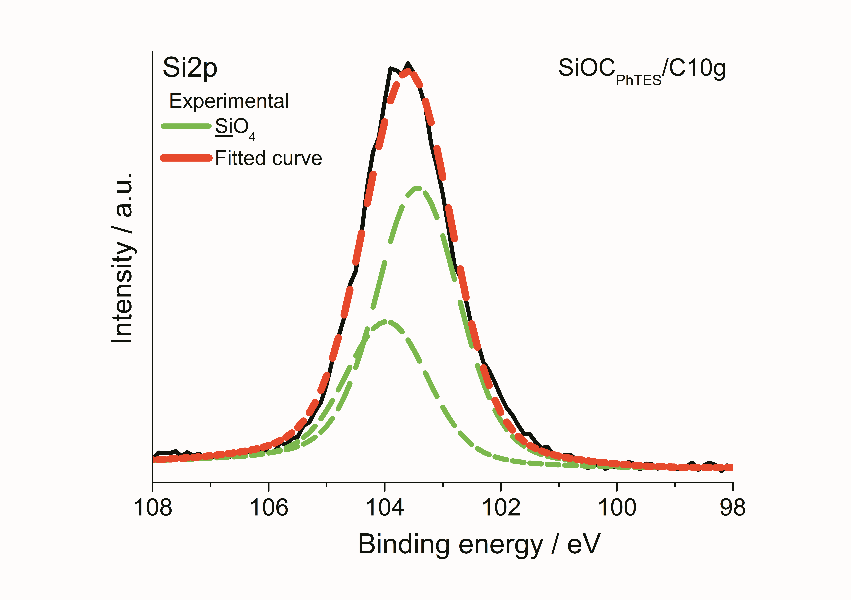  (**b**) |
| --- | --- |

**Figure S1.** XPS Si2p spectra of (**a**) SiOC_PhTES_ and (**b**) SiOC_PhTES_/C10g samples.

| 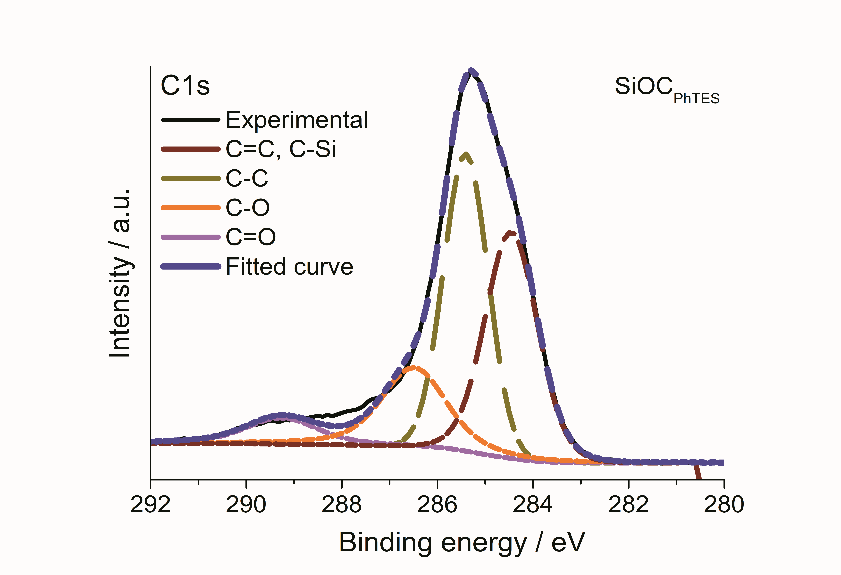  (**a**) | 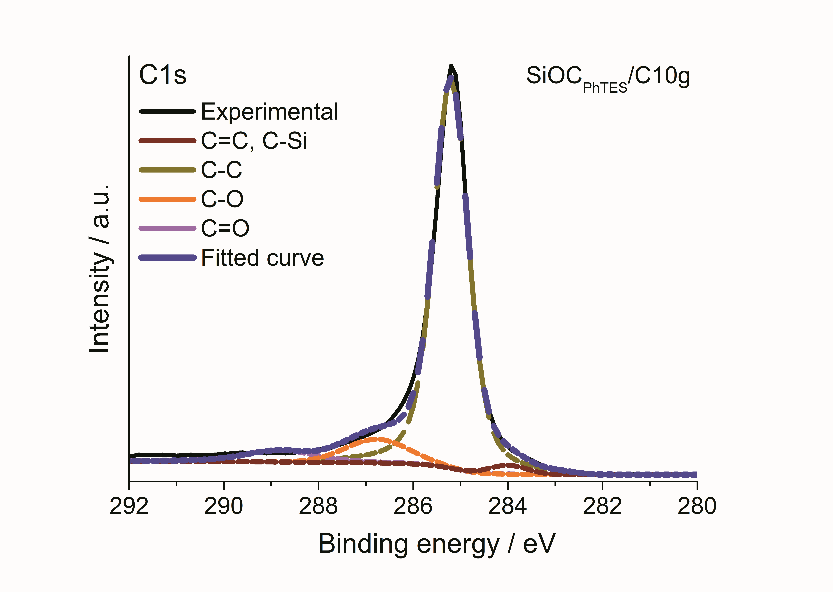  (**b**) |
| --- | --- |

**Figure S2.** XPS C1s spectra of (**a**) SiOC_PhTES_ and (**b**) SiOC_PhTES_/C10g samples..

| 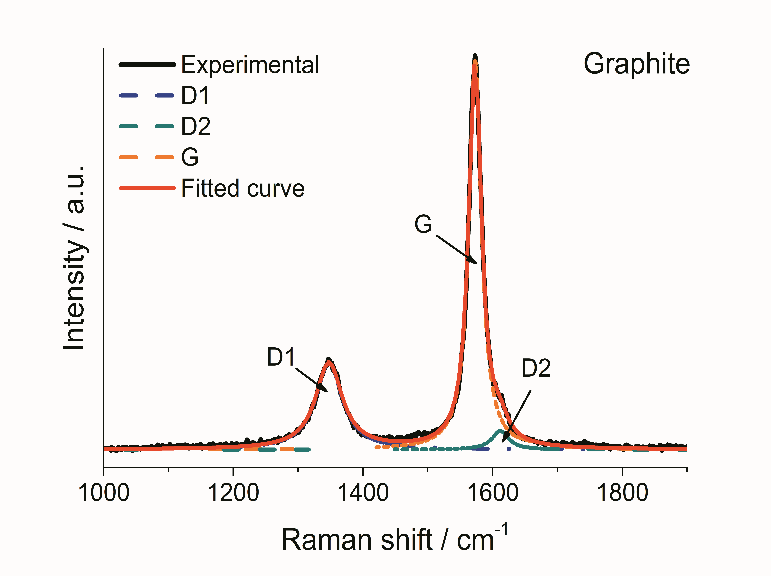  (**a**) | 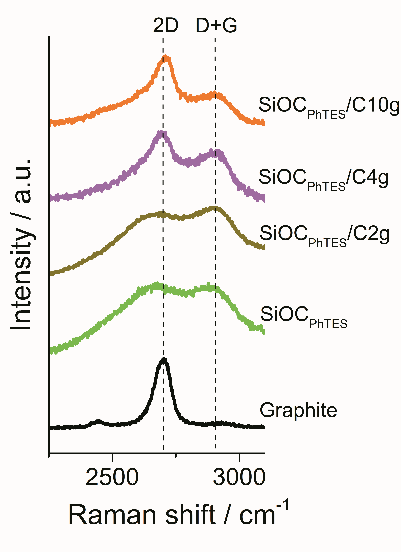  (**b**) |
| --- | --- |

**Figure S3.** (**a**) Deconvolution of Raman spectra of the pure graphite, (**b**) second-order Raman spectra of graphite, ceramic and SiOC/graphite composites.

**Table S1.** Data obtained from the deconvolution of Raman spectra.

| **Material** | **D4** | | | **D1** | | | **D3** | | | **G** | | | **D2** | | |
| --- | --- | --- | --- | --- | --- | --- | --- | --- | --- | --- | --- | --- | --- | --- | --- |
|  | **cm^-1^** | **Area int. %** | **FWHM** | **cm^-1^** | **Area int. %** | **FWHM** | **cm^-1^** | **Area int. %** | **FWHM** | **cm^-1^** | **Area int. %** | **FWHM** | **cm^-1^** | **Area int. %** | **FWHM** |
| SiOC_PhTES_ | 1194 | 7.07 | 213.73 | 1333 | 65.82 | 164.19 | 1525 | 7.61 | 114.24 | 1575 | 8.34 | 52.61 | 1608 | 11.17 | 43.63 |
| SiOC_PhTES_/C2g | 1193 | 4.04 | 159.73 | 1327 | 66.23 | 155.38 | 1528 | 7.41 | 135.00 | 1580 | 12.39 | 56.85 | 1609 | 9.93 | 35.85 |
| SiOC_PhTES_/C4g | 1195 | 5.72 | 219.73 | 1332 | 60.78 | 149.48 | 1525 | 7.62 | 143.96 | 1571 | 11.72 | 41.50 | 1603 | 14.16 | 48.20 |
| SiOC_PhTES_/C10g | 1197 | 6.90 | 188.33 | 1333 | 60.78 | 142.96 | 1528 | 7.97 | 131.37 | 1580 | 13.65 | 37.54 | 1610 | 10.71 | 38.59 |
| Graphite | - | - | - | 1348 | 30.62 | 49.83 | - | - | - | 1573 | 65.24 | 23.46 | 1611 | 4.14 | 31.20 |

| 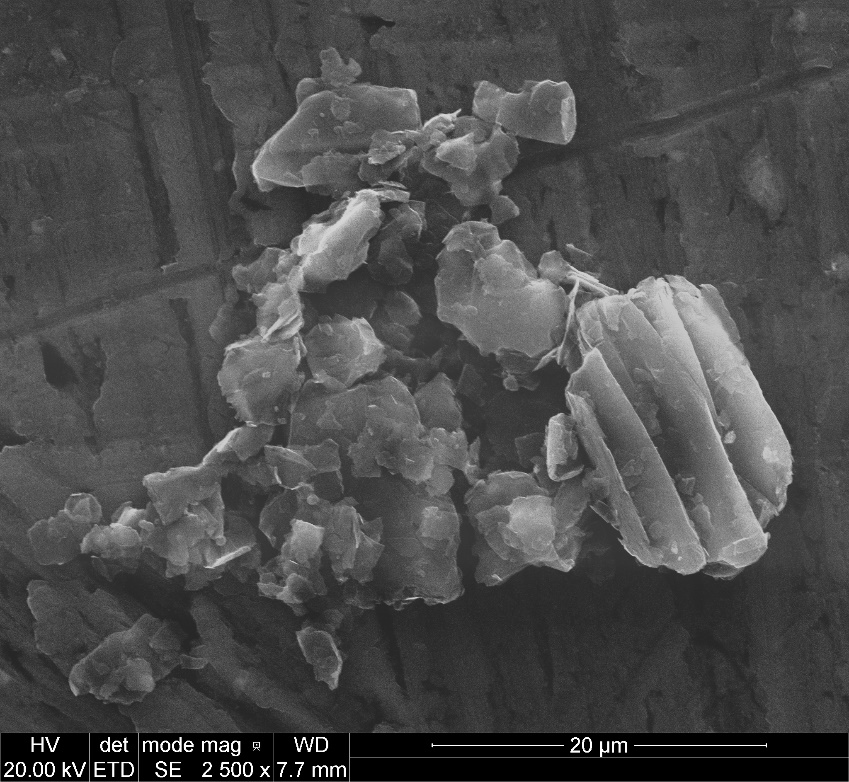  (**a**) | 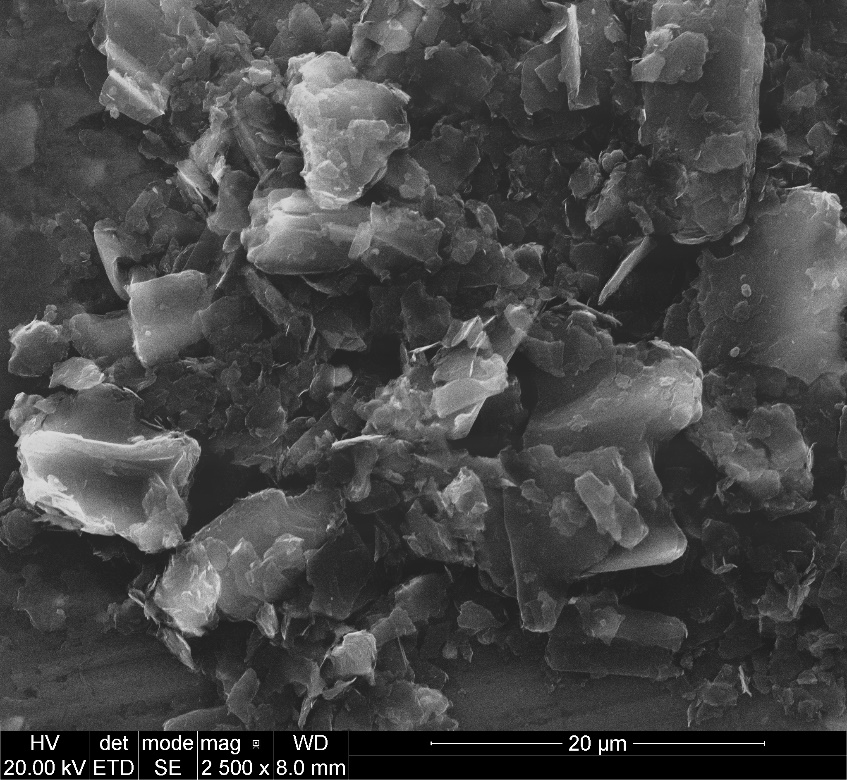  (**b**) |
| --- | --- |

**Figure S4.** SEM pictures of (**a**) graphite flakes, (**b**) graphite flakes after 2 h of sonication in isopropanol.
